# Mechanisms and Therapeutic Strategies for HCV/HBV-Associated B-Cell Non-Hodgkin’s Lymphomas: A Viewpoint

**DOI:** 10.32604/or.2025.071847

**Published:** 2026-02-24

**Authors:** Guido Carloni, Monica Rinaldi

**Affiliations:** Institute of Translational Pharmacology (IFT), Department of Biomedical Sciences (DBS), National Research Council (CNR), Rome, 00133, Italy

**Keywords:** B-cell non-Hodgkin’s lymphomas (B-NHLs), hepatitis C virus (HCV), hepatitis B virus (HBV), chronic infection, diffuse large B-cell lymphomas (DLBCL)

## Abstract

Hepatitis C virus (HCV) and hepatitis B virus (HBV) infections are increasingly recognized as significant etiological factors in the pathogenesis of B-cell non-Hodgkin’s lymphomas (B-NHLs). Epidemiological and molecular studies have demonstrated a consistent association between chronic viral infection and B-NHLs. Multiple pathogenic mechanisms have been implicated in lymphomagenesis, both direct and indirect, including chronic antigenic stimulation, direct infection of B cells, and viral protein–mediated oncogenic signaling, It is likely that a combination of several pathogenic conditions is required to eventually lead to the development of lymphoma. The prevalence of B-cell lymphomas among individuals with chronic HCV or HBV infection presents a complex pathogenetic scenario, given the tumor heterogeneity and variable clinical behavior, and poses therapeutic challenges, due to the partial efficacy of current treatment options. The advent of direct-acting antivirals (DAAs) for HCV and high-genetic barrier nucleos(t)ide analogues (NAs) for HBV has improved patient outcomes. In indolent HCV-associated B-NHLs, antiviral therapy with DAAs alone often achieves sustained virologic response and may lead to lymphoma regression. Conversely, aggressive subtypes like diffuse large B-cell lymphomas require combination treatment with immunochemotherapy. In the setting of HBV-associated lymphomas, antiviral prophylaxis with potent NAs (e.g., entecavir or tenofovir) is essential to prevent HBV reactivation during rituximab-containing chemotherapy regimen. The integration of antiviral and anticancer therapies has been shown to enhance survival outcomes while mitigating hepatic toxicity. A comprehensive understanding of the biological interplay between chronic viral infection and B-cell transformation is critical for optimizing diagnostic and therapeutic strategies. Aim of this viewpoint is to provide evidence that early viral detection and prompt management remain the most effective strategies to improve survival rates and to reduce treatment-related morbidity in these patients.

## 1 Introduction

Non-Hodgkin Lymphoma (NHL), one of the most common hematologic malignancies, comprises a heterogeneous group of lymphoid cancers. B-cell NHL accounts for approximately 85%–90% of all NHL cases. In 2021, NHLs represented 4.3% of malignant tumors, and 3.3% of cancer-related deaths in the USA.

The etiology of NHL remains largely unknown. Increasing evidence suggests that pathogen infections contribute to the pathogenesis of different subtypes of lymphoma. Approximately 15%–20% of NHLs are associated with a particular virus infection, including hepatitis C virus (HCV) and hepatitis B virus (HBV) [1–3]. This issue has clinical relevance, especially in countries with high prevalence of HBV and HCV infections.

In this viewpoint, we present a concise synthesis of current knowledge on HCV/HBV-associated B-cell NHLs, two highly complex and incompletely understood topics. This overview integrates up-to-date epidemiological, molecular and clinical data, while also discussing proposed pathogenetic mechanisms and therapeutic options.

Here, we aim to explore diagnostic and therapeutic strategies that may be useful to improve survival rates and to reduce treatment-related morbidity in these patients.

## 2 Association between HCV and B-NHL

The majority of individuals infected following exposure to HCV will develop chronic infection [4].

Extensive researches on the correlation between the HCV and B-cell non-Hodgkin’s lymphoma (B-NHL) initially have underlined a nearly 100% HCV seroprevalence in patients with mixed cryoglobulinemia (MC), a chronic lymphoproliferative disease [5,6]. Approximately 10% of MC patients are at risk of developing NHL, with a 35-fold increased risk of HCV-associated NHL compared to the general population [7]. Extensive investigations found an elevated risk of B-NHL in individuals with chronic HCV infection compared to uninfected individuals [8]. For istance, a study involving 150,000 HCV-infected patients in the USA found that HCV infection was associated with a 20%–30% increased risk of lymphoma [9]. Additional epidemiological data further support a marked increase in the risk of B-cell NHL among HCV-positive individuals [10]. Several investigations, including a meta-analysis, have shown that the prevalence of HCV infection in patients with B-cell NHL is ~15% [11–13]. Large, multicenter, case-control studies and epidemiological investigations have confirmed the causal association between HCV and B-NHL [11,14,15].

Successful anti-HCV therapy greatly reduces the risk of NHL among HCV patients. However, a sustained virological response (SVR)—defined as the absence of detectable HCV RNA 24 weeks after treatment was predominantly achieved in patients <65 years of age. This finding underscores the importance of early antiviral intervention in HCV-infected patients, [16]. Direct anti-HCV therapy triggers hematologic response in patients with indolent NHL and concurrent HCV infection, and is currently recommended as first-line treatment for asymptomatic indolent NHL [17].

Direct-acting antivirals (DAAs) have revolutionized the management of low-grade B-NHL, with about 50% of cases showing regression following therapy [18]. These interferon-free regimens achieve viral eradication in around 95% of patients within 8–12 weeks of treatment [19,20].

HCV infection is also associated with B-NHL subtypes that exhibit distinct genetic and clinical features, including diffuse large B-cell lymphoma (DLBCL), the most common aggressive NHL according to the WHO classification [21].

Patients with HCV-positive DLBCL generally exhibit higher International Prognostic Index (IPI) scores, worse overall survival (OS), and a significantly higher incidence of severe hepatic toxicity compared to their HCV-negative counterparts [22,23]. Poorer survival in these patients is linked to a combination of factors such as advanced disease, comorbidities, treatment intolerance, and liver-related toxicity. Liver fibrosis severity has been identified as a key prognostic factor in this group [24].

The optimal timing and approach to anti-HCV therapy in aggressive lymphoma remains uncertain. Standard immunochemotherapy with rituximab, cyclophosphamide, doxorubicin, vincristine, and prednisone/prednisolone (R-CHOP) is required for DLBCL.

Integrating DAAs before or concurrently with R-CHOP, rather than delaying antiviral treatment, is preferable to minimize the risk of toxicity due to high-dose immunosuppression [18].

DAAs have been demonstrated to safely and effectively reduce HCV viral load and improve outcomes in DLBCL patients [25]. A systematic review and meta-analysis examined the clinical characteristics and therapeutic responses to antiviral treatment and rituximab in NHL patients with HCV infection. HCV-positive NHL patients were found to have more advanced disease, higher frequency of liver and spleen involvement, shorter OS and progression-free survival (PFS), lower overall response rates (ORR), and higher rates of hepatic dysfunction during chemotherapy compared to HCV-negative patients.

Notably, antiviral therapy was associated with improved OS, reduced disease progression, and enhanced treatment response among HCV-infected NHL patients compared to those who did not receive antiviral therapy.

Furthermore, the inclusion of rituximab in treatment regimens resulted in significantly better OS. This meta-analysis provided compelling support for the combination of antiviral therapy with immunochemotherapy as an effective treatment strategy for HCV-positive NHL patients [26].

## 3 Association between HBV and B-NHL

The relationship between HBV infection and B-NHL has also been extensively investigated.

Evidence from HBV endemic areas have shown that the prevalence of HBV infection among NHL patients is considerably higher than in the general population.

Chronic HBV infection is defined as hepatitis B surface antigen (HBsAg) positivity. The risk of NHL, particularly the aggressive DLBCL, is significantly higher in HBsAg-positive (HBsAg+) groups compared with HBsAg-negative (HBsAg−) ones. A meta-analysis of 58 studies revealed that HBV infection confers a 2.5-fold increased risk of NHL. A real-world retrospective study reported a strong association between HBV infection and the development of B-cell NHL, and a possible role of HBV infection in the pathogenesis of B-NHL [27,28].

Multiple retrospective investigations and meta analyses have further identified DLBCL as the most prevalent NHL subtype among chronically HBV-infected patients [29,30].

HBV-associated DLBCL has been shown to have poor prognosis. Compared with HBsAg-DLBCL, the patients with chronic HBV infection tended to be diagnosed at a younger age, with more frequent splenic or retroperitoneal lymph node involvement, more advanced disease (stage 3 or 4) at diagnosis, and significantly worse outcomes [31,32]. The meta-analysis by Rong et al. corroborated these findings, demonstrating substantially lower 2-year and 5-year progression-free survival (PFS) and overall survival (OS) in patients with chronic HBV infection [33]. A more recent systematic review and meta-analysis by Zhang et al. provided additional insight into the clinical behavior of NHL in the context of chronic HBV infection. HBsAg-positive NHL patients showed consistently shorter OS and PFS, reduced complete remission rates, and a higher frequency of hepatic dysfunction during chemotherapy. They were also more likely to be younger at diagnosis, present with advanced disease, exhibit B symptoms, and show spleen or liver involvement. Analyses restricted to DLBCL mirrored these patterns [34].

Interestingly, even individuals who have recovered from prior HBV infection appear to have an elevated risk of developing B-cell lymphomas [35].

HBV reactivation (HBV-R) can occur years after the infection has been resolved [36]. Occult HBV infection is defined by the persistent presence of HBV DNA in serum or tissue despite the absence of detectable HBsAg. The stable maintenance of covalently closed circular DNA (cccDNA) within host cells serves as a template for viral gene transcription, contributing to occult infection. Because occult infection is more frequently detected in HBsAg-negative NHL patients, it has been proposed as a possible contributor to lymphoma onset and progression [37,38].

HBV infection also affects the chemotherapy effect and overall prognosis in NHL patients. HBsAg-positive DLBCL patients tipically show poorer responses to standard chemotherapy, with higher rates of progressive disease (PD) and lower rates of complete response (CR) than their HBsAg-negative counterparts [33].

Chemotherapy can concurrently activate HBV replication. This risk is particularly pronounced during rituximab-containing regimens (R-CHOP), where reactivation rates may reach 70%. Compared with CHOP alone, R-CHOP markedly increases the incidence of hepatic complications, and severe cases may progress to fulminant hepatitis [39,40].

Overall, accumulating evidence indicates that HBV-associated NHL is characterized by poor therapeutic outcomes and an unfavorable prognosis [41,42].

## 4 Pathogenesis of HCV-Associated B-NHL

HCV is a small, single-stranded RNA virus, consisting of an envelope and a nucleocapsid. The major components of the envelope are glycoproteins E1 and E2, which mediate viral internalization. E1 acts as a fusion protein during internalization, while E2 is responsible for binding to host cell receptors [43]. The CD81 receptor, crucial for HCV entry, plays a central role in B-cell infection.

Lymphotropic HCV strains can infect and replicate in B cells, T cells and dendritic cells (DC) cells through interaction with CD81 [44]. Infection of B cells via the CD21-CD19-CD81 complex can result in malignant lymphoma, while peripheral B cells can serve as a reservoir for HCV [45,46]. HCV infection and replication in T cells lead to reduced proliferative capacity, enhanced Fas-mediated apoptosis, and suppression of IFN-γ secretion [47,48]. As a result of HCV infection, alterations in apoptosis-related signaling pathways may contribute to T-cell hyporesponsiveness in some patients with hepatitis C. Both immature and mature dendritic cells are susceptible to HCV infection [49]. Chronic HCV infection is associated with an allostimulatory defect in DCs, which may serve as extrahepatic reservoir for the virus. DCs derived from chronically infected individuals show impaired T-cell stimulatory capacity due to altered expression of major histocompatibility complex (MHC) and costimulatory molecules on their surface [50,51].

Once internalized, the viral RNA is released and translated into structural (Core, E1, and E2) and non-structural proteins (p7, NS2, NS3, NS4A, NS4B, NS5A, and NS5) [52].

***Pathophysiological Processes***

**Figure 1:**
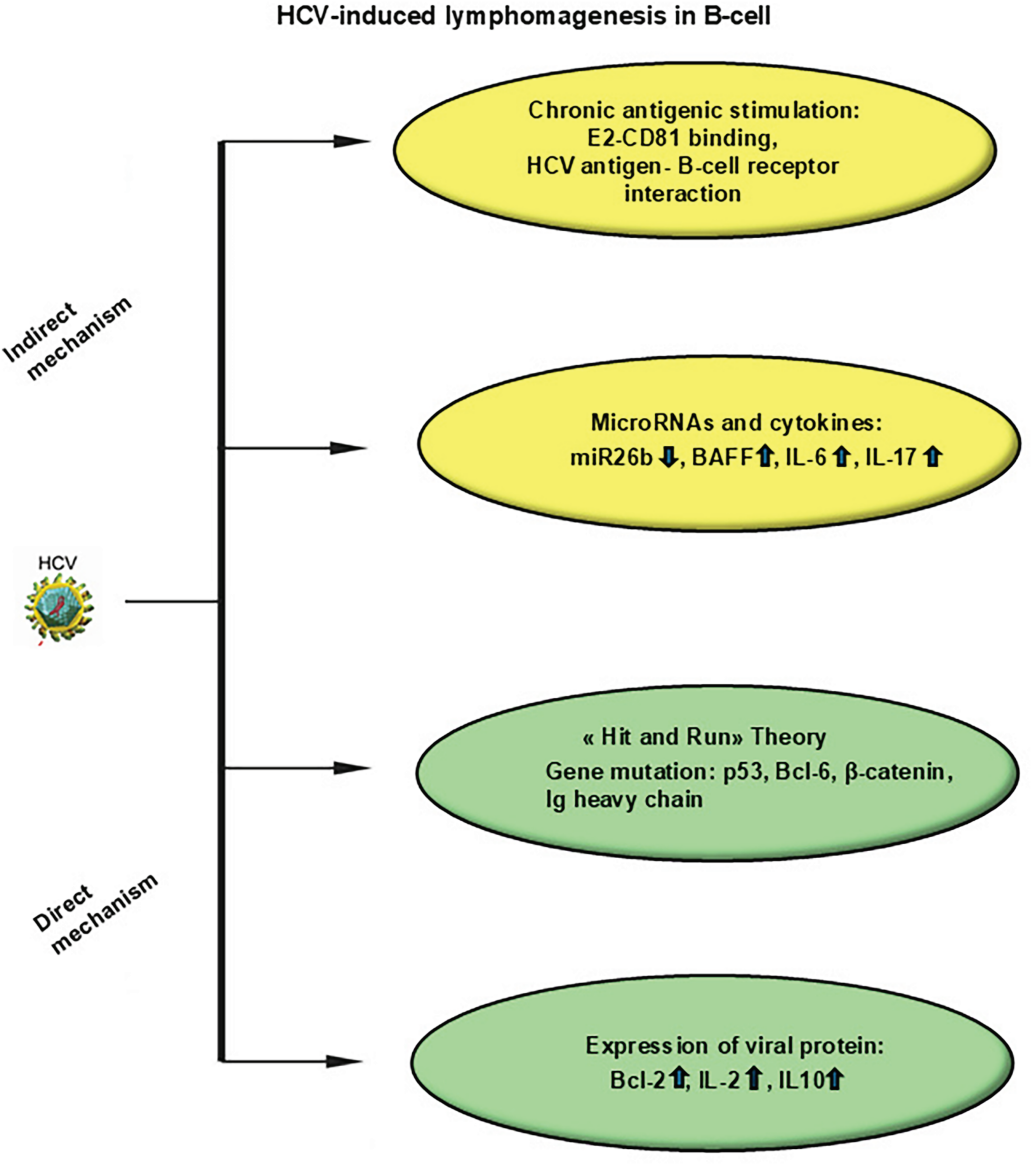
Mechanisms of HCV-induced lymphomagenesis in B-cells. Indirect mechanisms include chronic antigenic stimulation, microRNAs, cytokines. Direct mechanisms include “hit and run theory”, expression of viral proteins. Abbreviations: HCV, hepatitis C virus; IL, interleukin; BAFF, B-cell activating factor; BCL, B-cell lymphoma

The pathophysiological processes leading from HCV infection to overt lymphoma (schematically represented in Fig. 1) involve both indirect and direct mechanisms. Indirect mechanisms include chronic antigenic stimulation by extracellular HCV antigens and cytokines that drive monoclonal B-cell expansion. Direct mechanisms include oncogenic effects mediated by intracellular HCV proteins, particularly in DLBCL [53].

The interaction between HCV and B lymphocytes represents a central step in HCV-related lymphomagenesis. The viral envelope glycoprotein E2 binds to CD81, a tetraspanin broadly expressed on B cells. CD81 functions as a co-receptor of the B-cell receptor (BCR) complex and modulates antigen-driven signaling. Engagement of CD81 by E2 lowers the activation threshold of B cells, thereby favoring sustained proliferation and clonal expansion [54]. Beyond its co-stimulatory role, E2–CD81 interaction activates intracellular signaling cascades, including NF-κB, resulting in upregulation of anti-apoptotic proteins such as Bcl-2 and enhanced resistance to Fas-mediated cell death [55]. In addition to promoting B-cell survival, the E2–CD81 interaction contributes to genomic instability. Binding of E2 to B cells has been shown to induce double-strand DNA breaks within immunoglobulin (Ig) variable regions, a process linked to aberrant somatic hypermutation [56]. This phenomenon is further amplified by increased expression of activation-induced cytidine deaminase (AID), a key enzyme involved in antibody diversification. Dysregulated AID activity promotes mutations in Ig and non-Ig genes. Indeed, HCV-driven AID overexpression has been associated with mutations in oncogenes and tumor suppressor genes, including β-catenin, BCL6, and TP53, thereby facilitating malignant transformation of B cells [57].

Cytokines dysregulation represents an additional pathogenic pathway in HCV-associated lymphomas. Elevated levels of B-cell activating factor (BAFF), a cytokine critical for B-cell survival and maturation, have been observed in chronic HCV infection. BAFF levels are intermediate in patients with MC, and lowest in HCV-infected individuals without MC [58]. Experimental models further support a pro-tumorigenic role for BAFF, as BAFF-overexpressing transgenic mice develop lymphomas at high frequency, particularly in the absence of protective tumor necrosis factor signaling [59]. Other soluble mediators, including interleukins-6, -10, -17, and transforming growth factor-β (TGF-β), have also been implicated in sustaining B-cell activation and proliferation during chronic HCV infection.

A characteristic dysregulated microRNAs expression has been observed in splenic marginal zone lymphoma (SMZL), including miRNAs with previously recognized tumor suppressive or oncogenic potential, with a possible implication in molecular tumorigenesis [60].

Intracellular viral proteins additionally exert oncogenic effects through oxidative stress–related mechanisms. The HCV core and NS3 proteins can induce nitric oxide synthase (NOS) activity, leading to increased production of reactive nitrogen species. This oxidative milieu results in mitochondrial dysfunction, accumulation of DNA double-strand breaks, and an increased mutational burden within infected cells [61]. Consistent with this model, mutations affecting genes involved in cell fate determination and tumor suppression have been identified in HCV-positive DLBCL patients [62]. These observations support the so-called “hit-and-run” theory, whereby transient viral proteins expression induces permanent genetic alterations in B cells. Even after viral clearance, mutations in key regulatory genes—including TP53, BCL6, β-catenin, immunoglobulin heavy chain loci, and p53—may persist. Experimental studies have demonstrated a high mutation rate in HCV-infected B-cell lines and peripheral blood mononuclear cells, a finding corroborated *in vivo* in HCV-associated B-NHL.

Although the exact mechanisms of HCV leading to NHL has not been fully clarified, three main theories have been formulated to understand the HCV-induced transformation process. Clinical findings and experimental data strongly support the concept of continuous antigenic stimulation of lymphocytes by viral antigens leading to sustained proliferation. The concept of direct oncogenic effects by active replication of HCV in B cells mediated by intracellular viral proteins remains debated. Not all studies demonstrated the presence of HCV-RNA negative strands, the viral replicative intermediates, in human lymphocytes *in vivo*. Indeed, there is ample evidence that intracellular virus proteins could contribute to oncogenic transformation. The ‘‘hit and run’’ mechanism has been proposed to explain possible oncogenetic transformation of B cell (permanent B-cell damage). Doubts about the clinical relevance of this theory remain and *in vivo* findings are not always confirmed *in vitro* data.

Overall, current data support the idea that HCV lymphomagenesis is a complex, multistep, multifactorial process. It is driven by sustained B-cell activation, inhibition of apoptosis, and progressive accumulation of genetic aberrations in genetically predisposed background [63]. The increased frequency of NHL in MC patients supports the “Multistep Theory of HCV Lymphomagenesis” model.

## 5 Pathogenesis of HBV-Associated B-NHL

HBV is the prototype member of the Hepadnaviridae family and possesses a 3.2 kb partially double-stranded relaxed circular DNA (rcDNA) genome. The HBV genome contains four overlapping open reading frames (ORFs), four promoters, two enhancer elements (EN1 and EN2), a polyadenylation site for viral RNA transcription and several cis-acting signals for DNA replication. The ORFs P, S, C, and X encode DNA polymerase, the surface antigen (HBsAg), core and pre-core proteins, and X protein (HBx), respectively [64,65].

HBV initiates infection through low-affinity binding to heparan sulfate proteoglycans (HSPGs), preferentially glypican-5, on the host cell surface. HSPGs are widely distributed on human cells and within the extracellular matrix; however, the relative abundance of glypican-5 in hepatic tissue is thought to contribute to the hepatotropism of HBV.

HBV infects and replicates largely in hepatocytes where viral entry entry is mediated mainly through the sodium taurocholate cotransporting polypeptide (NTCP), which serves as the high-affinity receptor for the HBV large surface protein (L-HBsAg). Extrahepatic infection occurs through alternative mechanisms, which are less efficient and NTCP-independent. HSPGs mediate initial viral binding while auxiliary molecules, such as annexin V, fibronectin, and other surface glycoproteins, have been implicated in facilitating low-level entry or binding [66,67].

Once internalized, the viral capsid traffics to the nucleus, where rcDNA is released. The host DNA repair machinery then converts rcDNA into covalently closed circular DNA (cccDNA), highly stable in the nucleus of infected cells, which is packaged into chromatin by histone and non-histone proteins to form a viral minichromosome. This cccDNA serves as the transcriptional template for all viral mRNAs, including pregenomic RNA (pgRNA). The pgRNA is encapsidated with polymerase and reverse transcribed into viral minus strand DNA, followed by synthesis of the plus strand to form rcDNA. Mature nucleocapsids are either recycled to the nucleus to replenish cccDNA or enveloped and secreted as infectious virions [68].

Despite decades of research, key aspects of HBV replication—particularly cccDNA biogenesis and host pathways regulating its transcriptional activity—remain incompletely defined.

The specific role of HBV in the occurrence of NHL is still unclear, and is also less well characterized than that of HCV.

***Pathophysiological Processes***

**Figure 2:**
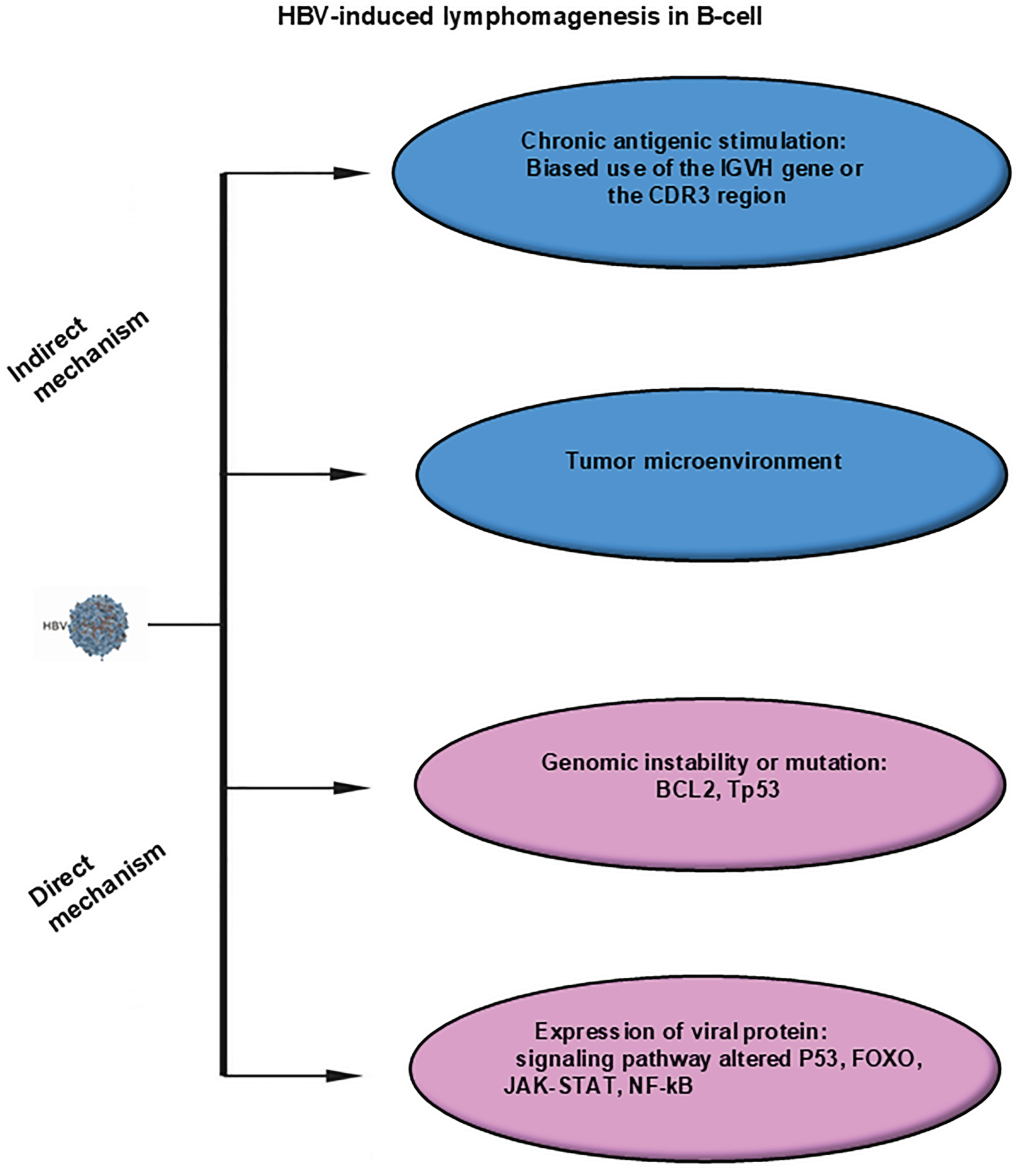
Mechanisms of HBV-induced lymphomagenesis in B-cells. Indirect mechanisms include chronic antigenic stimulation, tumor microenvironment. Direct mechanisms include genomic instability or mutations, expression of viral proteins. Abbreviations: HBV, hepatitis B virus; IGVH, immunoglobulin heavy chain variable region; CDR3, complementarity-determining region 3; TP53, tumor protein p53; FOXO, Forkhead box O; JAK, Janus kinase; STAT, signal transducer and activator of transcription; NF-κB, nuclear factor kappa-light-chain-enhancer of activated B cells

While the precise mechanisms linking HBV infection to lymphomagenesis remain incompletely defined, various hypotheses have been proposed to explain this association (schematically illustrated in Fig. 2).

The chronic antigen stimulation model seems less applicable to HBV-associated DLBC, respect to the antigen-driven HCV-associated lymphoma model. Current evidence supporting this mechanism is limited and inconsistent, warranting further investigation.

Conversely, more findings strongly support the idea that HBV infection of lymphocytes, through an overactive state of B cells, may result in enhanced mutations mediated in part by apolipoprotein B mRNA Editing Catalytic Polypeptide-like (APOBEC) and AID, leading to DLBCL. High-frequency mutations in HBV-positive DLBCL involve pathways associated with HBV infection, cell migration, immune evasion, epigenetic regulation, and major signaling pathways such as p53, FOXO, BCR, JAK-STAT, NF-κB [69,70].

HBV exhibits unique replication features involving RNA intermediates and the ability to integrate into the host genome [71]. Although primarily hepatotropic, HBV can directly infect B cells, where viral DNA integrates into the lymphocyte genome [72,73]. This integration, mediated by the reverse transcriptase activity of viral DNA polymerase, may directly activate pro-oncogenes or destroy tumor suppressors genes, leading to distinct pattern of genetic alterations, as observed in HBV-related hepatocellular carcinoma (HCC). Using a high-throughput virus integration assay, HBV DNA was also discovered to be integrated into preferential targets of NHL cell genome [74].

HBx protein, which is highly expressed in tissues from HBsAg positive DLBCL patients, might contribute to the malignant transformation and development of B cell NHL, as in HBV-induced HCC. However, the precise molecular mechanisms underlying HBx-mediated lymphomagenesis remain to be elucidated [75,76].

## 6 Treatment Strategies for HCV-Associated B-NHL

### 6.1 Antiviral Therapy (AT)

Successful regression of splenic lymphoma with villous lymphocytes following treatment of HCV infection with standard antiviral interferon (IFN)-α was first reported by Hermine and co-workers in 2002, suggesting a possible link between HCV infection and NHL [77]. Subsequently, several studies confirmed the efficacy of antiviral therapy (AT) in the management of HCV-associated NHL [78–80].

The regression of HCV-associated NHLs after viral eradication provides the strongest evidence supporting an etiological link between lymphoma and infection.

Advances in the understanding of HCV virology have led to development of new DAAs that target key components of virus replication. These include inhibitors of nonstructural (NS) proteins NS3/4A, NS5A inhibitors, nucleoside and non-nucleoside polymerase inhibitors [81].

NS5A replication complex inhibitors, exemplified by daclatasvir, represent a class of DAAs. NS5A is an attractive target because it is involved with multiple stages of the HCV life cycle. Due to their exceptional *in vitro* potency, broad genotype coverage and strong antiviral effects in infected patients, NS5A inhibitors are essential component of effective combination DAAs regimens. Based on drug-induced resistance substitutions and computational modeling, NS5A inhibitors are believed to act at the N-terminus of NS5A (domain I), thereby efficiently blocking both HCV RNA replication and virion assembly/secretion [82].

The HCV NS3/4A protease is a trypsin-like serine protease that plays a crucial role in generating components of the viral RNA replication complex. This protease cleaves the viral polyprotein downstream of the NS3 site, releasing functional proteins necessary for viral replication. In addition, it cleaves two cellular proteins involved in the interferon (IFN) signaling cascade, thereby blocking the host antiviral response. The antiviral activity of NS3/4A inhibitors, such as simeprevir, is mediated by non-covalent binding to viral protease, characterized by rapid association and slow dissociation kinetics [83].

Sofosbuvir, a nucleotide analog inhibitor, targets RNA NS5B polymerase, a key player in HCV replication. The active triphosphate form of sofosbuvir mimics the natural uridine nucleotide and is incorporated by the NS5B polymerase into the growing RNA chain, resulting in premature chain termination. SF is typically used in combination with other DAAs [84].

Different nucleoside inhibitors (NIs) and non-nucleoside inhibitors (NNIs) of NS5B viral RNA-dependent RNA polymerase have been developed to inhibit HCV replication. One advantage of the nucleoside inhibitors is their pan-genotypic effect. They target the active site of HCV polymerase and generated resistant mutants are replication defective, thereby providing a high barrier to the emergence of resistant mutants. In contrast, NNIs target allosteric sites on the polymerase, and their efficacy varies among genotypes; consequently, they present a lower barrier to the emergence of resistant variants [85].

The development of resistant viral mutants remains the principal cause of antiviral treatment failure. The barrier to resistance varies across the classes of drugs being used for HCV. A major challenge for the development of drug resistance in HCV patients is the high genetic diversity of the viral genome which supports the production of large viral variants during infection and selection of resistant variants during treatment. Drug resistance to DAAs is common with monotherapy regimens. To predict the optimal number and combination of drugs required to minimize resistance, mathematical modeling approaches have been employed [86]. Different studies showed that combination therapy with DAAs yields superior clinical outcomes and significantly reduces the likelihood of resistance development [85–87]. Thus, several DAAs have been approved for use in various IFN-free combination regimens.

The introduction of DAAs has revolutionized the treatment of HCV infection, achieving viral eradication rates approaching 100% across nearly all genotypes [87,88]. DAA-based regimens promote sustained virologic response (SVR) rates of 90%–100%, thus demonstrating excellent efficacy in eradicating HCV infection [89,90].

Only a few studies have reported complete lymphoproliferative disease response (LDR) of the hematological disease following DAAs treatment [91]. In the prospective study conducted by Merli and colleagues, all patients achieved sustained virologic response (100%). However, the overall lymphoma response rate was 45%, including eight patients (20%) with complete response and ten (25%) with partial response [88].

### 6.2 Anti-Cancer Chemotherapy

DAAs administration is considered the first-line therapy for patients with indolent lymphomas who do not require immediate cytoreductive treatment [81,91,92]. In contrast, antiviral therapy alone is unlikely to be sufficient for aggressive HCV-related NHL. More aggressive subtypes, such as DLBCL, typically require the addition of DAAs to standard anti-cancer chemotherapy (CHOP), often in conjunction with the B-cell-depleting anti-CD20 antibody rituximab (R-CHOP). This antibody targets the CD20 transmembrane receptor on B cells, leading to rapid depletion of circulating CD20^+^ B cells.

Immunochemotherapy (I-CT) has become the standard first-line approach for NHL, particularly DLBCL, the most common histological subtype [93]. Clinical studies in HCV-positive DLBCL patients have demonstrated favorable outcomes when antiviral therapy is integrated into lymphoma management, including high SVR rates, acceptable tolerability, and improved progression-free survival.

Concurrent administration of DAAs and R-CHOP appears preferable to deferred administration of DAAs for the potential prevention of hepatic toxicity, which has been reported to be as high as 27% during R-CHOP administration [94]. Concomitant DAA treatment during chemotherapy has been shown to be safe and effective in promoting remission of aggressive lymphomas in HCV-infected patients. No significant difference was observed in OS after 52 weeks between DAA-treated and untreated patients; however, a statistically significant improvement in disease-free survival (DFS) was achieved in patients receiving DAAs [95].

## 7 Treatment Strategies for HBV-Associated B-NHL

Several studies have demonstrated that HBV infection may adversely affect OS and PFS in patients with DLBCL treated with R-CHOP chemotherapy, compared with HBV negative individuals [33,96,97]. Virological serum markers have been shown a strong impact on OS and PFS among patients with concurrent HBV infection and DLBCL undergoing R-CHOP therapy [98]. Notably, Hepatitis B envelope Antigen (HBeAg)-positive patients exhibited a trend toward inferior OS and PFS compared with HBeAg-negative patients. Conversely, anti-HBe-positive patients demonstrated significantly improved OS and PFS compared with anti-HBe–negative counterparts. High-level viremia (HBV-DNA ≥ 2 × 10^7^ IU/L) was also associated with significantly worse OS and PFS outcomes [99].

### 7.1 Immuno-Chemotherapy

In contrast, HBV infection poses a significant clinical challenge during lymphoma treatment due to the risk of viral reactivation. Accumulating evidence indicates that HBV-R is strongly associated with cytotoxic chemotherapy and B-cell-depleting therapies, particularly anti-CD20 monoclonal antibodies such as rituximab [100]. HBsAg-positive patients receiving immunosuppressive treatment are at substantial risk of reactivation, commonly defined by a ≥100-fold increase in serum HBV DNA levels [101].

Moreover, lymphoma patients with resolved HBV infection are at high risk of HBV reactivation when treated with rituximab or other potent immmunosuppressive agents [102]. In these patients, HBsAg seroconversion or an increase in serum HBV DNA concentrations usually indicate HBV reactivation. The presence of antibodies against HBsAg (anti-HBs) significantly reduces the risk of reactivation compared with patients who are anti Hepatitis B core (anti-HBc)-positive only [103].

HBV-R may result in severe hepatitis, liver failure, and increased mortality if not promptly recognized and treated [104]. The risk of HBV-R is influenced by both host-related and treatment-related factors. Virus-related determinants include baseline HBV DNA levels, HBsAg and HBcAb positivity, viral genotype, and mutations affecting the HBsAg region [105]. Patients who are HBsAg-positive are at the highest risk, whereas those with protective anti-HBs antibodies and high titers have the lowest risk [106]. Treatment-related risk is largely determined by the intensity and type of immunosuppression. The R-CHOP regimen remains the standard first-line therapy for DLBCL, but its immunosuppressive components significantly increase the likelihood of HBV-R. Risk categories range from very high (>20%) with anti-CD20 antibodies, to high (10%–20%) with anthracycline-based regimens or prolonged high-dose corticosteroids, to moderate (1%–10%) and low (<1%) risk with less intensive therapies [101,107–109]. Rituximab induces prolonged B-cell depletion through complement-mediated and antibody-dependent cytotoxicity and has been shown to impair immunity. Its use is associated with delayed cytopenias, hypogammaglobulinemia, and an increased risk of late HBV reactivation [110]. Anthracyclines, such as doxorubicin, further contribute to HBV-R by directly enhancing viral transcription and replication [101,111,112].

### 7.2 Antiviral Therapy

HBV-related NHL hence requires an appropriate choice of clinical management. All NHL patients should undergo HBV screening (at least HBsAg and HBcAb testing) before initiation of immunosuppressive therapy. Baseline HBV DNA quantification should be measured for patients positive for either HBsAg or HBcAb. A panel of HBV serologic markers, including HBsAg, anti-HBs, HBeAg, anti-HBe, and anti-HBc should be assessed in all at-risk patients, prior to chemotherapy or intensive immunosuppression, particularly in those from HBV-endemic areas. Antiviral prophylaxis should be strongly considered at least one week before the start of immunosuppressive therapy in HBsAg-positive or HBcAb-positive patients, regardless of baseline viral load, due to their high risk of HBV-R.

Current clinical guidelines recommend antiviral therapy for all patients who are HBsAg-positive with HBV DNA levels ≥2000 IU/mL. Although immunocompetent individuals with lower viral loads may not routinely require treatment, those scheduled to receive immunosuppressive or cytotoxic therapy should initiate prophylactic antiviral treatment prior to chemotherapy [113]. For patients at moderate or low risk of HBV-R, a pre-emptive strategy—initiating antiviral therapy at the earliest sign of rising HBV DNA—may be considered appropriate [104].

Several nucleos(t)ide analogues (NAs) including entecavir (ETV) and tenofovir, effectively inhibit HBV reverse transcriptase and DNA replication and are widely used in the management of HBV-R in NHL patients. The long-term use of lamivudine (LMV) is associated with a high incidence of drug resistance, particularly beyond one year of therapy. Consequently, newer-generation NAs such as ETV and tenofovir are now preferred due to their high genetic barrier to resistance and superior antiviral potency, as demonstrated in multiple studies such as clinical study [114], meta-analyses [115–117], retrospective cohort study [118] and prospective study [119].

The optimal duration of antiviral prophylaxis remains debated. Rituximab-induced immunosuppression may persist for more than one year after the last administration [101,120], and HBV reactivation has been reported even more than two years after completion of rituximab-containing regimens [121].

Current evidence suggests that antiviral prophylaxis should continue for at least six months after cessation of immunosuppressive therapy, and for at least 12 months in patients receiving B-cell-depleting agents such as rituximab [122]. Given the profound and prolonged immunosuppressive effects of rituximab, lifelong antiviral therapy may be the most appropriate option for the majority of patients at persistent risk [123].

## 8 Conclusions

The considerable prevalence of B lymphomas in HCV/HBV-infected subjects has significant clinical implications and poses challenges in accurate diagnosis, risk assessment, and therapeutic decision-making.

From a clinical perspective, several key recommendations emerge:

(1) Routine viral screening (HBsAg, anti-HBc, anti-HBs, and HCV RNA) should be performed in all newly diagnosed B-NHL patients, regardless of liver enzyme status or geographic region;
(2) HCV-associated indolent B-cell NHLs should receive DAA therapy as first-line treatment, which can achieve effective viral clearance and lymphoma regression in approximately half of patients;
(3) Aggressive HCV-associated lymphomas (e.g., DLBCL) should be managed with concurrent or sequential DAA therapy combined with standard immunochemotherapy (R-CHOP) to optimize outcomes and minimize hepatotoxicity;
(4) HBV-positive patients (HBsAg+ or anti-HBc+) must receive prophylactic antiviral therapy (with entecavir or tenofovir) prior to rituximab-containing regimens and continue treatment for at least 12 months after of therapy completion—or longer in cases of persistent immunosuppression;
(5) Monitoring of HBV DNA and liver function is mandatory during and after immunochemotherapy, even in patients with resolved infection (occult HBV);
(6) Close collaboration between hematologists, hepatologists, and infectious disease specialists is crucial to ensure safe and effective management of viral-driven lymphomas.

More structured detailed recommendations, highly relevant and helpful for clinicians, are provided in the recently updated ECIL 9 (ECIL-9, Lancet Haematol. May 2025) Clinical Practice Guidelines for the management of HBV and HCV infections in patients with hematological malignancies. This document offers comprehensive, evidence-based recommendations on prevention, screening, treatment, and long term surveillance, with the contribution of a group of experts in the fields of viral hepatitis and of haematological malignancy. Recommendations and statements on screening of hepatotropic viruses before haematological treatment and of patients with markers of past or current viral hepatitis, on vaccination, as well on treatment rules, are reported in this document.

In this viewpoint, we sintetized current knowledge on HCV and HBV-associated B-cell non-Hodgkin lymphomas. We reported a subset of the evidence and theories available in the literature regarding the pathogenetic mechanisms, which represents a limitation of this study. Overall, the integration of antiviral therapy with modern immunochemotherapy has revolutionized the therapeutic paradigm for HCV/HBV-associated B-NHL, offering the prospect of both viral eradication and durable lymphoma control.

## 9 Future Perspectives

For HCV-associated indolent NHL, DAA therapy is recommended. Preliminary evidence suggests that early initiation of anti-HCV treatment may offer a preventive benefit in the development of B-cell NHL. Furthermore, hepatitis B vaccination has been linked to a reduced incidence of NHL. Thus, expanding access to antiviral therapies and promoting widespread vaccination efforts could play a critical role in reducing the burden of virus-associated malignancies. Emerging treatment strategies are currently under development. A deeper understanding of the immune system may enhance therapeutic outcomes through checkpoint blockade approaches, bispecific antibodies, small targeted molecules and engineered T-cell immunotherapies. Chimeric Antigen Receptor T-cell (CAR-T) therapies have significant potential in the treatment of patients with viral-associated lymphomas. Currently, a phase I trial is underway to evaluate the safety and feasibility of using CAR cells for treating HBV-NHL.

## Data Availability

Not applicable.
